# Superoxide Dismutase Prevents SARS-CoV-2-Induced Plasma Cell Apoptosis and Stabilizes Specific Antibody Induction

**DOI:** 10.1155/2022/5397733

**Published:** 2022-01-17

**Authors:** Li-Hua Mo, Xiang-Qian Luo, Ke Ma, Jian-Bo Shao, Guo-Hao Zhang, Zhi-Gang Liu, Da-Bo Liu, Huan-Ping Zhang, Ping-Chang Yang

**Affiliations:** ^1^Department of Pediatric Otolaryngology, Shenzhen Hospital, Southern Medical University, Shenzhen, China; ^2^Guangdong Provincial Key Laboratory of Regional Immunity and Diseases, Shenzhen, China; ^3^Institute of Allergy & Immunology, Shenzhen University School of Medicine, State Key Laboratory of Respiratory Disease Allergy Division at Shenzhen University, Shenzhen, China; ^4^Department of Allergy Medicine, Bethune Hospital Affiliated to Shanxi Medical University, Taiyuan, China; ^5^Department of Otolaryngology, Head and Neck Surgery, Beijing Children's Hospital, Capital Medical University, National Center for Children's Health (NCCH), Beijing, China; ^6^Department of Otolaryngology, Guangzhou Women and Children's Medical Center, Guangzhou Medical University, Guangzhou, China

## Abstract

The infection of coronavirus disease (COVID-19) seriously threatens human life. It is urgent to generate effective and safe specific antibodies (Abs) against the pathogenic elements of COVID-19. Mice were immunized with SARS-CoV-2 spike protein antigens: S ectodomain-1 (CoV, in short) mixed in Alum adjuvant for 2 times and boosted with CoV weekly for 6 times. A portion of mice were treated with Maotai liquor (MTL, in short) or/and heat stress (HS) together with CoV boosting. We observed that the anti-CoV Ab was successfully induced in mice that received the CoV/Alum immunization for 2 times. However, upon boosting with CoV, the CoV Ab production diminished progressively; spleen CoV Ab-producing plasma cell counts reduced, in which substantial CoV-specific Ab-producing plasma cells (sPC) were apoptotic. Apparent oxidative stress signs were observed in sPCs; the results were reproduced by exposing sPCs to CoV in the culture. The presence of MTL or/and HS prevented the CoV-induced oxidative stress in sPCs and promoted and stabilized the CoV Ab production in mice in re-exposure to CoV. In summary, CoV/Alum immunization can successfully induce CoV Ab production in mice that declines upon reexposure to CoV. Concurrent administration of MTL/HS stabilizes and promotes the CoV Ab production in mice.

## 1. Introduction

Since the beginning of 2020, COVID-19 infection has been a global pandemic disease that seriously threatens human life and has caused a catastrophic consequence for healthcare systems worldwide [1]. The clinical phenomena of COVID-19 mainly include fever, cough, interstitial pneumonia, acute distress syndrome, and eventually, multiorgan failure [2]. At the beginning of COVID-19 pandemic, management of COVID-19 infection mainly focuses on supportive therapy, symptom control, and preventing respiratory failure [1]. Currently, vaccines against COVID-19 have been employed in the general population to prevent COVID-19 infection. The efficacy of this vaccination has to be verified in further investigation.

There are many research groups working on the development of vaccines against SARS-CoV-2 for the prevention and treatment of COVID-19 [1, 3, 4]. Using the SARS-CoV-2 viral spike protein (CoV, in short) as an antigen, the anti-SARS-CoV-2 antibody (Ab) has been successfully developed [1, 3, 4]. The Ab can neutralize SARS-CoV-2 and prevent the virus from binding the receptor, angiotensin-converting enzyme 2 (ACE2). Similar to others [1, 3, 4], we induced the anti-CoV Ab in mice. However, we observed that the Ab production declined quickly in mice after the immunization. Further experimental evidence showed apoptotic activities in the CoV-specific Ab-producing plasma cells (sPCs). Therefore, we carried out this project. The results showed that reexposure to CoV could induce reactive oxygen species (ROS) and decrease superoxide dismutase (SOD) in sPCs. Upregulating the SOD production prevented the reexposure to CoV-induced sPC apoptosis and dramatically prolonged the CoV Ab production.

## 2. Materials and Methods

### 2.1. Mice

Male C57BL/6 (8-10-week-old) mice were purchased from Guangdong Experimental Animal Center (Guangzhou, China). ACE2-deficient mice were purchased from Jackson Laboratory (Bar Harbor, ME). Mice were maintained in a specific pathogen-free facility and allowed to access water and food freely. The experimental procedures were approved by the Animal Ethical Committee at Shenzhen University.

### 2.2. Specific CoV Ab Generation in Mice

Following our established procedures [5], C57BL/6 mice were immunized with SARS-CoV-2 spike protein antigens: S ectodomain-1 (CoV, in short; 50 *μ*g/mouse) mixed in 0.1 ml Alum through subcutaneous injection in the back skin in week 1 and week 2, respectively. From week 3, mice were boosted by subcutaneous injection with CoV (20 *μ*g/mouse) weekly for 6 consecutive weeks (from week 3 to week 8; Fig. S1A in supplemental materials).

### 2.3. Enzyme-Linked Immunosorbent Assay (ELISA)

The levels of IL-4, IL-6, IFN-*γ*, TNF-*α*, ROS, MDA, and SOD in the serum or culture supernatant were determined by ELISA with commercial reagent kits following the manufacturer's instruction. To assess the CoV Ab levels in the serum, microplates (96 wells) were coated with 100 ng/100 *μ*l of CoV in PBS and incubated overnight at 4°C. After blocking with 1% BSA (bovine serum albumin) for 30 min, serum samples (diluted 1 : 5) or CoV Ab (used to create standard curves) were added to the plates (100 *μ*l/well) and incubated for 1 h at room temperature. Serum samples from naive mice were used as controls. The plates were washed with PBST (PBS containing 0.05% Tween 20) 3 times; bound antibodies were detected by incubating with an HRP-labeled anti-mouse IgG Fc-antibody for 1 h and washed with PBST 3 times. Tetramethylbenzidine (TMB) was added to plates and incubated for 10 min. Absorbance was recorded at 450 nm. The results were calculated against the standard curve.

### 2.4. Competitive ELISA

In *CoV Ab preparation*, blood samples were collected from mice after complete immunization. The serum was isolated from the blood and passed through a CoV-adsorbed sepharose column. The CoV Ab was eluted with an eluting buffer.

In *HRP-CoV preparation*, CoV was labeled with HRP using an HRP-labeling kit (Sigma Aldrich) following the manufacturer's instruction.

In *competitive mixture preparation*, HRP-CoV was mixed with CoV Ab (0-10000-folds in a 10-fold downgradient dilution) for 30 min at room temperature.

In *competitive ELISA*, microplates (96 wells) were coated with 100 ng/100 *μ*l of recombinant ACE2 proteins in PBS and incubated overnight at 4°C. After blocking with 1% BSA (bovine serum albumin) for 30 min, a mixture of HRP-CoV/CoV Ab was added to each well (100 ng/100 *μ*l HRP-CoV). The following procedures are the same as regular ELISA as shown above.

### 2.5. Detection of CoV Ab Producing Plasma Cells (sPCs)

The CoV protein antigen was labeled with FITC (using a FITC-labeling reagent kit purchased from Sigma-Aldrich following the manufacturer's instruction). After staining with CD138 and B220 Abs, spleen cells were fixed with 1% paraformaldehyde (containing 0.05% Triton X-100) for 1 h at room temperature and washed with PBS 3 times; the cells were stained with FITC-CoV (100 ng/ml) or isotype IgG for 30 min at 4°C and washed with PBS 3 times. The cells were analyzed with flow cytometry (FACS).

### 2.6. Treating Plasma Cells with MTL

MTL was added to plasma cell culture medium equal to 1% alcohol. Controls were treated with saline or 1% ethanol.

### 2.7. Treating Plasma Cells with Heat Stress (HS)

Plasma cells were treated with HS by placing the culture vials (sealed by a plastic wrap) in a water bath with temperature adjusted to 37, 40, 42, or 45°C, respectively, for 1 h.

### 2.8. Treating Mice with MTL

Following published procedures [6], mice were gavage-fed with MTL (5 ml/kg of 53% MTL) together with each boosting of CoV.

### 2.9. Treating Mice with HS

Following published procedures [7] with a minor modification, mice were placed in a specific incubator (Isotemp-Fisher Scientific Incubator, Dubuque, IA) at 40°C for 1 h at each boosting of CoV. The sham HS group mice were placed in a similar incubator at room temperature.

P.S., reagents, mice, preparation of single spleen cells, flow cytometry, detection of apoptotic plasma cells, isolation plasma cells from the spleen, cell culture, RNA sequencing, plasma cell protein extraction, Western blotting, ROS detection by flow cytometry in plasma cells, coimmunoprecipitation, detection of SOD ubiquitination, mass spectrometry analysis, and statistics are presented in the supplementary materials.

## 3. Results

### 3.1. Anti-CoV Ab Production Declines during Extending Immunization

**Figure 1:**
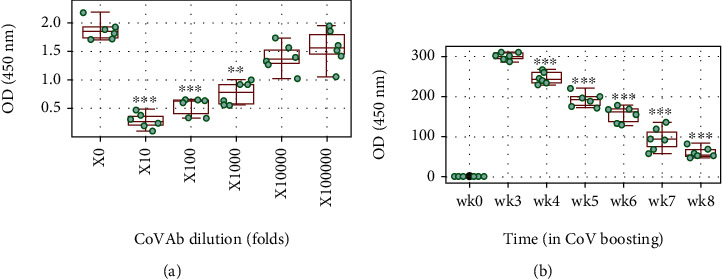
Assessment of serum CoV-specific Ab levels during boosting with CoV. C57BL/6 mice were immunized with CoV as the specific Ag. (a) Boxplots show induced CoV Ab/ACE2 binding test results. (b) Boxplots show serum CoV-specific Ab (CoV-sAb) levels during immune boosting (wk: week). Statistics: ANOVA followed by Dunnett's test. ^∗∗^*p* < 0.01, compared with group 0 (a). ^∗∗∗^*p* < 0.001, compared with group 0 (a) or wk3 (b). Each group consists of 6 mice in (b). The data of boxplots are presented as median (IQR). Each dot in boxplots presents data obtained from one sample (in triplicate).

Published data indicate that the anti-CoV Ab is inducible in animal experiments as well as in a human being that has therapeutic potential to be employed in controlling CoVID-19 [3, 4]. In line with those pioneer studies, we also induced anti-CoV Ab in mice that could efficiently block the binding between CoV and ACE2 (Figure 1(a)). We employed an established immunization protocol of our laboratory [5] to generate CoV-Ab in mice that consisted of immunization 2 times, followed by boosting 6 times, once a week. High CoV-Ab levels were detected in the serum in week 3. However, the serum CoV Ab levels were diminished progressively from week 4 to week 8 (Figure 1(b)).

**Figure 2:**
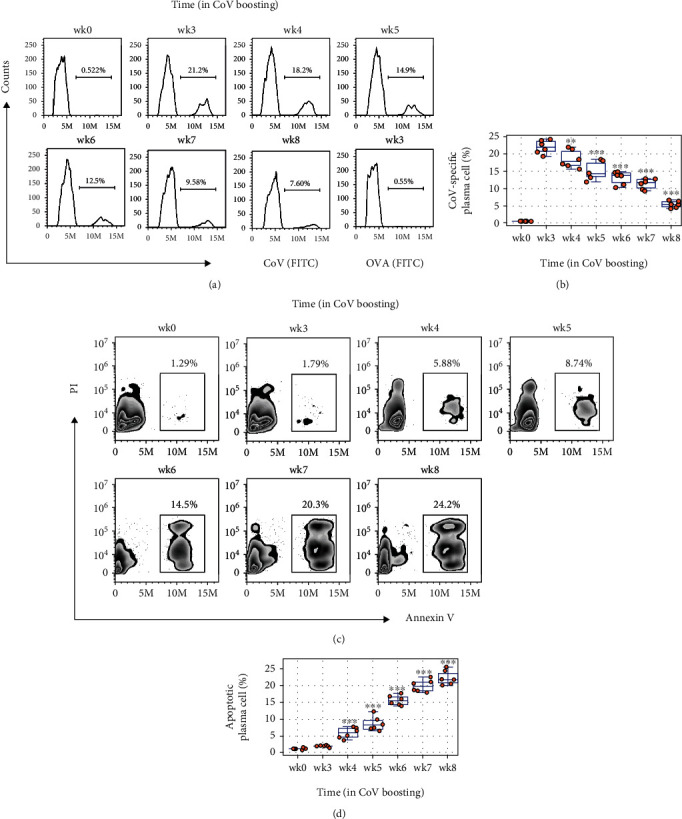
Assessment of CoV-specific plasma cell apoptosis during boosting with CoV. (a) Spleen cells were prepared from CoV-immunized and boosted mice at the indicated timepoints. Gated histograms show CoV Ab-positive spleen plasma cells (from gated plasma cells as shown in Fig. S1. The last panel (labeled in blue) shows OVA does not bind sPCs. (b) Boxplots show summarized CoV Ab-positive plasma cells. (c, d) Gated FACS plots show apoptotic portion in CoV-specific plasma cells. Violin plots show summarized apoptotic plasma cell counts. ^∗∗∗^*p* < 0.001, compared with group wk3. Statistical methods: ANOVA followed by Dunnett's test. Each group consists of 6 mice. Each dot in violin plots presents data obtained from one mouse.

We then took an insight into the mechanism of CoV Ab diminishing. Since plasma cells are responsible for Ab production, we assessed plasma cells in the mouse spleen. The results showed that the frequency of total spleen plasma cells did not apparently changed during immunization (Fig. S1 in supplemental materials). We then assessed the specific CoV-Ab-producing plasma cells (sPC) by staining cells with fluorescence-labeled anti-CoV Ab and assessed by FACS. The results showed that the sPC frequency were 0.34% ± 0.1% (mean ± SEM) in wk0 (week 0), 21.9% ± 2.1% in wk3, 18.6% ± 2.1% in wk4, 14.2% ± 2.4% in wk5, 11.7% ± 2.2% in wk6, 10.6% ± 1.6% in wk7, and 5.5 ± 1.1% in wk8, respectively (Figures 2(a) and 2(b)). The results indicate that sPC diminish progressively in mice in parallel to the times of CoV boosting. This prompted us to assess plasma cell apoptosis. Indeed, apoptotic spleen sPCs were detected by FACS: 1.2% ± 0.12% in wk0, 1.9% ± 0.23% in wk3, 5.8% ± 1.6% in wk4, 9.7% ± 2.5% in wk5, 15.1% ± 1.3% in wk6, 20.1% ± 1.7% in wk7, and 22.1% ± 1.7% in wk8 (Figures 2(c) and 2(d)). The results suggest that two times of immunization with CoV results in high CoV Ab production in mice, while extending exposure to CoV induces sPC apoptosis that results in diminishing the sPC number and thus diminishing CoV Ab production.

### 3.2. Exposure to CoV Induces sPC Apoptosis

**Figure 3:**
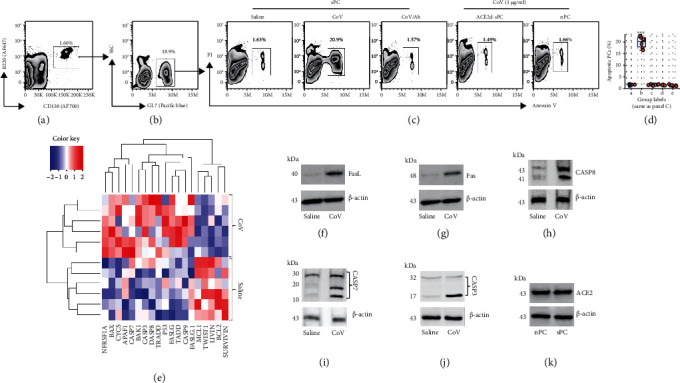
Assessment of CoV-exposure-induced sPC apoptosis. (a, b) Plasma cells were isolated from CoV-immunized mouse spleen by FACS and cultured in the presence of CoV (1 *μ*g/ml) for 24 h to activate sPC (a, b). (c) sPC, ACEd-PC, and nPC were exposed to CoV or saline in the culture for 24 h as denoted above each subpanel. Gated FACS plots show apoptotic sPC frequency. ACE2d: ACE2-deficient plasma cells (from ST2-deficient mice); nPC: plasma cells were isolated from the naïve mouse spleen; CoV/Ab: CoV and CoV Ab were mixed at a ratio of 1 : 1 before adding to the culture. (d) Violin plots show summarized apoptotic sPC counts. (e) sPCs were analyzed by RNAseq. Heatmap shows apoptotic related gene activities in plasma cells. The data of each row were obtained from one sample. (f–j) sPC protein extracts were analyzed by Western blotting. Immunoblots show indicated protein levels. (k) Immunoblots show plasma cells express ACE2, the CoV receptor. ^∗∗∗^*p* < 0.001 (ANOVA followed by the Dunnett's test), compared with group A. The group labels in (b) are the same as those in (a). The data represent 6 independent experiments.

The data of Figures 1 and 2 suggest that extending exposure to CoV (the boosting procedures) may induce sPC apoptosis. To verify this, we assessed the effects of CoV exposure on inducing sPC apoptosis. Plasma cells were isolated from spleen cells of mice immunized with CoV by FACS with CD138 and B220 as the cell markers. Plasma cells were exposed to CoV in the culture for 24 h and analyzed by FACS. The results showed that exposure to CoV in the culture did induce sPC, but not naïve PC (nPC), apoptosis. Blocking CoV by mixing CoV with CoV Ab before adding to the culture abolished the effects of CoV on inducing sPC apoptosis (Figures 3(a)–3(d)). We also observed that exposure to CoV for 24 h markedly increased the gene activities (Figure 3(e)) and increased protein amounts of caspase (CASP) 8, CASP7, CASP3, Fas, and FasL in sPCs (Figures 2(f)–2(j)). In addition, ACE2, the receptor of CoV, was detected in sPC and nPC (Figure 2(k)). The results demonstrate that exposure to CoV induces sPC apoptosis.

### 3.3. Exposure to CoV Increases ROS and Decreases SOD in CoV Ab-Producing Plasma Cells

**Figure 4:**
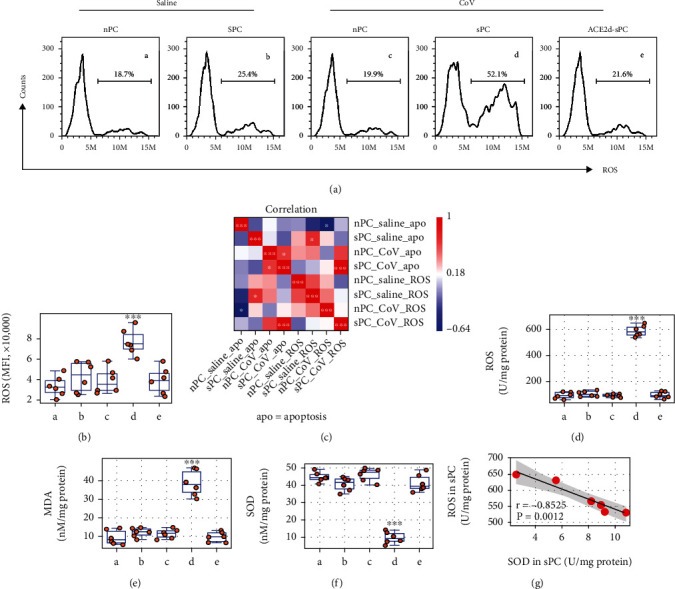
CoV increases ROS in plasma cells. nPC and sPC were prepared and exposed to CoV (10 *μ*g/ml) in the culture for 48 h. (a) The gated FACS histograms show ROS in plasma cells. (b) The bars show the mean fluorescence intensity (MFI) of ROS staining in plasma cells. (c) The heatmap shows positive correlation between plasma cell MFI and the CoV-induced plasma cell apoptosis (the data are presented in Figure 3). (d–f) Boxplots show levels of ROS, MDA, and SOD levels in plasma cells. (g) Scatter plots show negative correlation between SOD and ROS in sPCs. ACE2d: ACE2-deficient plasma cells. ^∗∗∗^*p* < 0.001 (ANOVA followed by Dunnett's test), compared with group A. The data of boxplots are presented as median (IQR). Group labels of (b–f) are the same as those in (a). Each dot in boxplots presents data obtained from one sample. The group labels of (b–f) are the same as (a).

We then took an insight into the mechanism by which CoV induces plasma cell apoptosis. Since reactive oxygen species (ROS) is one of the causative factors inducing apoptosis [8], we analyzed the ROS levels in plasma cells by FACS after CoV exposure. We found that exposure to CoV in the culture for 48 h, the ROS levels in plasma cells were markedly increased (Figures 4(a) and 4(b)), which were positively correlated with the apoptotic plasma cell frequency (Figure 4(c)). The results were verified by ELISA that showed exposure to CoV significantly increased the levels of ROS and MDA (malondialdehyde), as well as decreased the superoxide dismutase-1 (SOD, in short) levels in sPCs (Figures 4(d) and 4(f)). A negative correlation was detected between ROS levels and SOD levels in sPCs (g). Depletion of ACE2 in plasma cells abolished the CoV-induced changes of ROS, MDA, and SOD in plasma cells (Figure 4). The results demonstrate that exposure to CoV results in oxidative stress in sPCs, which is positively associated with sPC apoptosis.

### 3.4. Exposure to CoV Suppresses SOD Expression in sPCs through the JAK2/STAT1 Pathway

**Figure 5:**
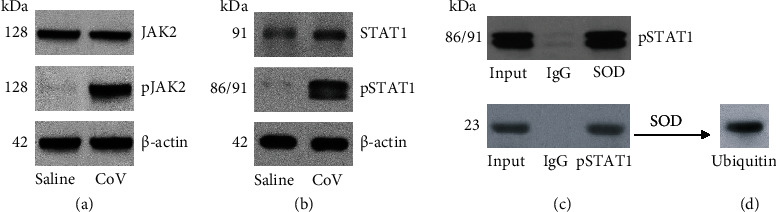
CoV exposure activates the JAK2/STAT1 signal pathway in sPCs. sPCs were prepared and exposed to CoV in the culture at 10 *μ*g/ml overnight. (a, b) Protein extracts of sPCs were analyzed by Western blotting. Immunoblots show phosphor JAK2 and STAT1. (c) co-IP results show a complex of pSTAT1/SOD in sPC protein extracts. (d) Ubiquitin was colocalized at the SOD plots in (c) (by reblotting on the same membrane). The data are from one experiment that represents 6 independent experiments.

The data of Figures 3 and 4 indicate that exposure to CoV induces plasma cell apoptosis and suppresses SOD in plasma cells. As the Janus kinase (JAK)2/STAT1 (signal transducer and activator of transcription 1) pathway activation is associated with cell apoptosis initiation [9], the JAK2/STAT1 activation in sPCs was assessed after exposing to CoV overnight. The results showed that CoV exposure markedly induced the JAK2 and STAT1 phosphorylation in sPCs (Figures 5(a) and 5(b)). By reviewing the STAT1 amino acid sequence in NCBI database, we realized that the STAT1 molecule might bind other proteins to form complexes during the CoV exposure since there are 5 PxxP structures in the amino acid sequence (Fig. S2 in supplementary materials) that can be designated the proline-rich proteins. Proline-rich proteins are associated with the SH3 domains that have been found in many protein molecular structures of signal transduction pathways and tend to adhere to other proteins [10]. Using an anti-pSTAT1 Ab as a bait, they presumed that pSTAT1 complexes were precipitated from protein extracts of CoV-treated plasma cells. The complexes were analyzed by mass spectrometry analysis. We found that the pSTAT1 formed a complex with SOD (Fig. S3). This was verified by coimmunoprecipitation (co-IP; Figure 5(c)). Since the SOD amounts in plasma cells were decreased after exposure to CoV, we inferred that the physical contact between pSTAT1 and SOD might trigger or facilitate the SOD degradation. Ubiquitination is an important step in protein degradation, in which ubiquitin attaches target proteins to facilitate the protein degradation [11]. To this end, we assessed ubiquitin in the protein complexes of pSTAT1/SOD in CoV-primed plasma cells. Indeed, the results showed a high ubiquitin amount in the complexes of pSTAT1/SOD (Figure 5(d)). The data indicate that CoV exposure suppresses SOD in plasma cells through activating the JAK2/STAT1 signal pathway.

### 3.5. Regulating the ACE2/JAK2/STAT1 Signal Pathway Blocks the Specific Antigen Exposure-Induced Plasma Cell Apoptosis

**Figure 6:**
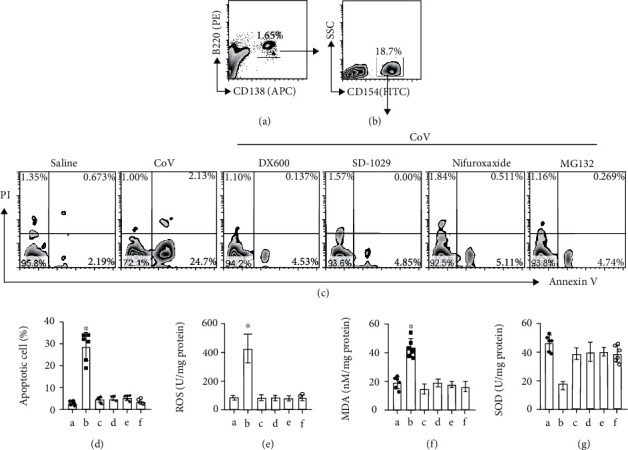
Regulation of the ACE2/JAK2/STAT1 signal pathway blocks the CoV exposure-induced sPC apoptosis. sPCs were prepared (a, b) and treated with the agents denoted above each subpanel of (a) for 48 h. CoV: 10 *μ*g/ml. DX600 (an ACE2 inhibitor): 10 *μ*g/ml. SD-1029 (a JAK2 inhibitor): 100 nM. Nifuroxazide (an inhibitor of multiple STATs): 20 *μ*M. MG132 (an inhibitor of proteasome and ubiquitin): 10 *μ*M. (c) Gated FACS plots show apoptotic sPC counts. (d) Bars show summarized apoptotic sPC counts. (e–g) Bars show levels of ROS, MDA, and SOD in sPCs. ^∗^*p* < 0.01 (ANOVA followed by Dunnett's test), compared with group A. The group labels in (d–g) are the same as those in (c). The data represent 6 independent experiments.

The data of Figure 5 implicate that exposure to CoV may activate the ACE2/JAK2/STAT1 signal pathway that eventually induce sPC apoptosis. To test this, sPCs were isolated from CoV-immunized mouse spleen and exposed to CoV in the culture for 48 h with or without the presence of inhibitors of JAK2 or STAT1 or ubiquitin or ACE2, respectively. The sPCs were prepared and analyzed by FACS (Figures 6(a) and 6(b)). We found that exposure to CoV-induced sPC apoptosis that was blocked by the presence of the inhibitors of either JAK2 or STAT1 or ubiquitin or ACE2, respectively (Figures 6(c) and 6(d)). The presence of inhibitors also suppressed ROS and MDA levels as well as enhanced SOD levels in CoV-treated sPCs (Figures 6(e)–6(g)). The results demonstrate that the ACE2/JAK2/STAT1 signal pathway involves the CoV exposure-induced sPC apoptosis.

### 3.6. Upregulating the SOD Levels in Plasma Cells by Heat Stress and Maotai Liquor Promotes the Anti-CoV Ab Induction

**Figure 7:**
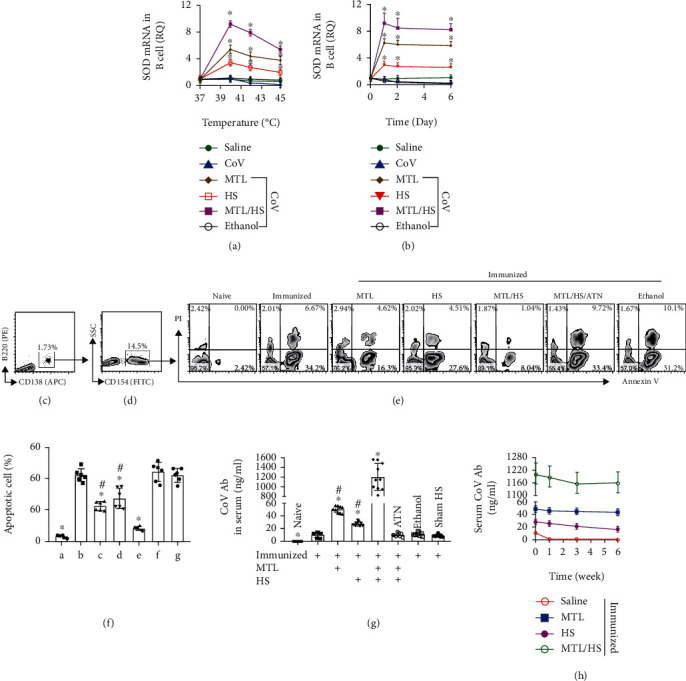
Regulating SOD expression in plasma cells promotes anti-CoV Ab induction. (a–f) PCs were isolated from the spleen of immunized mice and treated with additional conditions in the culture as denoted in the figures. CoV: 10 *μ*g/ml. MTL: 1% Maotai liquor (contains 1% alcohol) in culture medium. HS: heat stress by placing cell culture vials in a water bath with indicated temperature for 1 h. Ethanol: 1%. Curves indicate SOD mRNA levels in sPCs (a, b). (c, d) sPCs were gated from the whole PCs. (e) Gated FACS plots show apoptotic sPC counts (Annexin V^+^ or Annexin V^+^ PI^+^). ATN: ATN224 (a SOD1 inhibitor; 3.5 *μ*M). (f) Bars show summarized apoptotic sPC counts from 6 experiments per group. (g) Mice were immunized with CoV/Alum and boosting with CoV weekly for 6 weeks, and the additional conditions denoted in the figure. MTL: mice were gavage-fed with MTL (5 ml/kg of 53% MTL) together with each CoV boosting. HS: mice were treated with HS at each CoV boosting. ATN: mice were treated with ATN224 (4 mg/kg; i.p.) every other day in the entire experimental period. Ethanol: Mice were gavage-fed with ethanol (5 ml/kg of 53% ethanol). Sham HS: Mice were treated with sham HS. Bars show serum CoV-specific Ab levels. (h) Curves show serum CoV Ab levels in mice after CoV immunization and CoV boosting. ^∗^*p* < 0.01, compared with the saline group (a, b, h) or with group B (f) or with the immunized alone group (g). ^#^*p* < 0.01, compared with the MTL/HS group (h). Statistical methods: ANOVA followed by Dunnett's test (a, b) or Bonferroni's test (f–h). Each group of (g, h) consists of 10 mice.

The data of Figures 3–6 indicate that exposure to CoV decreases SOD levels in sPC that is associated with sPC apoptosis after CoV exposure. The data suggest that upregulating the SOD expression in plasma cells may stabilize the anti-CoV antibody production. Referring to published data that heat stress (HS) upregulates SOD production in *E*. *coli* [12] and Maotai liquor (MTL; a Chinese alcohol-containing beverage) suppresses ROS and MDA and increases the SOD production in liver cells [6], we treated sPCs with HS or/and MTL in the culture. The results showed that treating sPCs with 1% MTL or HS [13] at 40°C markedly increased SOD expression in sPCs that diminished at 42°C and dropped below the baseline at 45°C (Figure 7(a)). The concomitant presence of both MTL and HS resulted in an even higher increase in the SOD expression in sPCs. The effects of upregulating SOD expression in sPCs by MTL or/and HS slightly reduced next day but kept at high levels at least for 6 days, in which the concurrent exposure to both MTL and HS achieved the best efficiency in stabilizing SOD expression in sPCs (Figure 7(b)). MTL or/and HS (40°C) also efficiently prevented the exposure to CoV-induced sPC apoptosis, in which the presence of both MTL and HS achieved even better effects on the apoptosis inhibition that was abolished by the presence of ATN224, an inhibitor of SOD1 [14] (Figures 7(c)–7(f)). We then expanded the cell culture study finding in an animal model study by immunizing mice with CoV/Alum with or without the supplement of MTL gavage and treatment with HS following published procedures [6] [7]. The mice were sacrificed after the 8 wk immunization/boosting. Serum anti-CoV Ab levels were detectable in the CoV alone group that were markedly increased in the CoV/MTL group and the CoV/HS group; treating with CoV and both MTL and HS achieved even higher anti-CoV Ab levels in the serum that were 120.78 times over the CoV alone group, 24.15 times over the CoV/MTL group, and 43.84 times over the CoV/HS group, respectively. The anti-CoV Ab production was not statistically altered in the ethanol control group or in the sham HS group (Figure 7(g)). Moreover, we also observed that the serum CoV Ab kept at high levels after the 8 wk immunization/boosting for at least 6 additional weeks as we assessed the Ab levels at weeks 1, 3, and 6, respectively, after completing the 8 wk immunization/boosting (Figure 7(h)). Additionally, the experimental mice looked healthy, no body weight loss (Fig. S4) and no significant changes in serum inflammatory cytokine levels (Fig. S5). The results demonstrate that upregulating the SOD expression in plasma cells can markedly promote the anti-CoV Ab induction, which can be achieved by the administration of MTL supplement plus HS. On the other hand, the total number of spleen PCs was not significantly altered after CoV immunization with or without the additional treatment with MTL or/and HS (Fig. S6, S7). PCs were detectable in the bone marrow; the total PC number in the bone marrow was also not significantly altered by CoV immunization (data not shown).

## 4. Discussion

COVID-19 seriously threatens human life. It is urgent to develop effective and safe remedies to be used in the prevention and treatment of COVID-19 infection. Our research group also participated in this important work to induce anti-SARS-CoV-2 Ab with a murine model. In line with published data [1, 3, 4], we induced anti-CoV Ab in mice with CoV as the specific antigen. However, the Ab production did not last long in mice. The reason of this phenomenon was that exposure to CoV increased the ROS and decreased the SOD levels in sPCs and induced sPC apoptosis. This phenomenon was prevented by administration of the MTL supplement and HS.

The present study showed that immunizing mice with CoV and Alum adjuvant successfully induced anti-CoV Ab in mice. This is supported by two datasets. One is that the experimental results are processed by ELISA, indicating that the induced Ab can bind CoV, one of the components of the SARS-CoV-2 virus; others also used this antigen to generate specific Ab against SARS-CoV-2 [3]. Another dataset shows that the CoV Ab can competitively bind CoV to prevent CoV Ab from binding ACE2. ACE2 is the receptor of the SARS-CoV-2 virus. The virus binds this receptor for entry. Blocking this receptor can be a therapeutic option [15]. We used Alum as the adjuvant to generate the CoV Ab. Alum is a mild immune adjuvant. Using the complete Freund adjuvant is another option that may achieve better Ab generation [16].

We observed that the CoV-specific Ab production declined in mice one week after the first two times of immunization. Similar phenomena were also found by others; e.g., Zhang et al. reported that the generated specific Ab against SARS-CoV F69 strain decreased significantly one week after the peak value [16]. The present study revealed the underlying mechanism of this phenomenon that the number of sPCs reduced spontaneously in mice, of which more than 70% sPCs show annexin V positive. Annexin V can bind phosphatidylserine on the cell surface. The increase in phosphatidylserine on the cell surface is an indicator of cell apoptosis or the cells are going to apoptosis [17]. Our data suggest that plasma cell apoptosis may be the reason for the CoV Ab declining in mice.

By *in vitro* cell culture experiments, we found that exposure to CoV in the culture-induced sPC apoptosis. The data show that apoptosis initiating molecules, including CASP8, CASP7, CASP3, Fas, and FasL, were markedly upregulated in sPCs by CoV exposure. These apoptosis-related molecules are associated with the mitochondrial apoptosis pathway [18], suggesting that the oxidative stress-related apoptosis pathway may be activated in sPCs upon reexposure to CoV. Oxidative stress is one of the factors inducing cell apoptosis [8]. Our data also show that CoV exposure increased ROS and MDA levels and reduced the SOD levels in sPCs. The data also show that CoV exposure increased STAT1 phosphorylation; the latter facilitates protein degradation by promoting protein ubiquitination [19]. By the stripping-reblotting approach, high ubiquitin levels were colocalized in the complex of pSTAT1/SOD. This provides mechanistic evidence to support the results that CoV exposure markedly suppressed the SOD levels in sPCs.

By modulating the ACE2/JAK1/STAT1 signal pathway, we successfully manipulated the CoV exposure-induced sPC apoptosis, increase in ROS/MDA, and decrease in SOD in sPCs. This suggests that CoV exposure induced the sPC apoptosis is regulatable. Published data indicate that MTL and HS can upregulate SOD expression [6, 12]. By employing MTL and HS in the experiments, the expression of SOD was significantly increased in sPCs that resulted in preventing the CoV-induced sPC apoptosis and stabilizing the CoV Ab production. MTL is an alcohol-containing beverage, but its possible role of the alcohol in the regulation of sPC apoptosis and CoV Ab production can be ruled out since the ethanol control group did not show such a benefit. Published data show that MTL can counterpart oxidative stress in liver cells by increasing SOD production and inhibiting ROS and MDA [6]. MTL also contains SOD (6667 U/100 ml) and manganese (0.022 mg/100 ml); those are important for alleviating the oxidative stress injury [20]. Thus, it is of significance to fractionate the substances to induce SOD in sPCs from MTL that may be conducted in the future studies. The data also show that treating mice with mild HS is beneficial to generating and stabilizing CoV Ab. Similar to employing MTL, HS also increased SOD and suppressed ROS/MDA in sPCs and promoted CoV Ab production in mice. Privalle and Fridovich explain that this effect of HS is to disrupt the electron transport assemblies of the plasma membrane and thus increase the Mn SOD biosynthesis [12]. Importantly, the combination of MTL supplement and HS dramatically promoted and stabilized CoV Ab production in mice. The MTL supplement and HS used in the present study did not result in apparent body injury in mice, nor induced systemic inflammatory response.

It should be mentioned that, in the present study, mice were immunized with CoV/Alum 2 times and boosting with CoV 6 times, while in current human COVID-19 vaccination, only two times are required in general. It seems that the 6-time boosting is unnecessary. However, it revealed an important factor that somehow mimics reexposure to COVID-19 virus after immunization. The results suggest that after receiving COVID-19 vaccination, sPCs were induced in the body. The sPCs may become apoptosis upon reexposure to COVID-19. The present data suggest that such a phenomenon can be prevented by relevant remedies, such as MTL and HS, as tested in the present study.

In summary, the present study found that CoV immunization could induce CoV Ab production in mice, but the Ab production declined upon reexposure to CoV. The underlying mechanism of this phenomenon was CoV exposure-induced oxidative stress in sPCs and induced sPC apoptosis that could be prevented by employing the MTL supplement and HS.

## Data Availability

All the data are included in this paper and the online supplemental materials.

## Supplementary Materials

Supplementary Materials Supplemental materials include reagent information, Maotai liquor description, preparation of single spleen cells, flow cytometry, detection of apoptotic specific plasma cells, isolation plasma cells from the spleen, cell culture, RNA sequencing (RNAseq), plasma cell protein extraction, Western blotting, ROS detection by flow cytometry in plasma cells, coimmunoprecipitation (co-IP), detection of SOD ubiquitination, mass spectrometry analysis, and statistics, supplemental Figures S1-S7 and the raw data of RNAseq.
