# Supplementary material for: Superoxide Dismutase Prevents SARS-CoV-2-Induced Plasma Cell Apoptosis and Stabilizes Specific Antibody Induction
Source: Oxid Med Cell Longev. 2022 Jan 17;2022:5397733. doi: 10.1155/2022/5397733 (PMC8762758; doi:10.1155/2022/5397733)
Supplement: Supplementary Materials — Supplemental materials include reagent information, Maotai liquor description, preparation of single spleen cells, flow cytometry, detection of apoptotic specific plasma cells, isolation plasma cells from the spleen, cell culture, RNA sequencing (RNAseq), plasma cell protein extraction, Western blotting, ROS detection by flow cytometry in plasma cells, coimmunoprecipitation (co-IP), detection of SOD ubiquitination, mass spectrometry analysis, and statistics, supplemental Figures S1-S7 and the raw data of RNAseq. [file 5397733.f1.docx]

**Supplementary materials**

**Reagents**

SARS-CoV-2 spike protein and antibody were purchased from GenScript Biotech (Nanjing, China). c ELISA kit was purchased from Bioss Biomart (Beijing, China). GL7 Ab (Pacific blue) was purchased from Biolegend (San Diego, CA). ACE2 RNAi kits, antibodies (Abs) of Fas (clone#: G-9), FasL (NOK-1), (ACE2 (E-11), SOD (B-1), JAK2 (C-10), caspase (CASP)8, CASP3 (4.1.18), CASP7 (10-1-62), B220 (RA3-6B2; PE, AF647), and SD-1029, nifuroxazide, HRP-labeled anti-mouse IgG Fc antibody, were purchased from Santa Cruz Biotech (Santa Cruz, CA). Abs of FasL (ab15285), pJAK2 (E132), STAT1 (ab239360), pSTAT1 (phospho S727, ab109461) and ubiquitin (ab134953) were purchased from abcam (Cambridge, MA). APC and AF700-CD138 Ab (281-2) was purchased from BD Biosciences (Franklin Lakes, NJ). ELISA kits of ROS, MDA, SOD, IL-4, IL-6, IFN-γ and TNF-α were purchased from Biocompare (South San Francisco, CA). CellROX™ Green Flow Cytometry Assay Kit was purchased from Thermo Fisher Scientific (Burlington, ON, Canada). Recombinant ACE2 was provided by the Sangon Biotech (Shanghai, China). Annexin V kit, TMP, FITC-labeling kit were purchased from Sigma Aldrich (St. Louis., MO). Materials and reagents for RT-qPCR and Western blotting were purchased from Invitrogen (Carlsbad, CA).

**Maotai liquor**

The Maotai liquor was purchased in Maotai Liquor Airport Store (This store is owned by Maotai Liquor Manufacturer in Zunyi, China). The alcohol level is 53%. The properties of Maotai Liquor can be found in literature, such as (1) Zhou W, Chen Z, Zhang G, Liu Z. Systems pharmacology-based approach for dissecting the mechanisms of pyrazine components in Maotai liquor. Biosci Rep. 2019 Oct 30; 39(10): BSR20191864, (2) Wang W, Liu R, Shen Y, Lian B. The Potential Correlation Between Bacterial Sporulation and the Characteristic Flavor of Chinese Maotai Liquor. Front Microbiol. 2018 Jul 2; 9: 1435, and (3) Wu ZY, Lei XJ, Zhu DW, Luo AM. Investigating the Variation of Volatile Compound Composition in Maotai-Flavoured Liquor During Its Multiple Fermentation Steps Using Statistical Methods. Food Technol Biotechnol. 2016 Jun;54(2):243-249.

**Preparation of single spleen cells**

Mouse spleens were excised, cut into several pieces. The tissues were grinded into single cells in a cell strainer. Red blood cells were lysed with an ACK lysis buffer. Spleen cells were filtered through a cell strainer. Cell viability was greater than 99% as checked by the Trypan blue exclusion assay.

**Flow cytometry (FACS)**

To detect plasma cells, spleen single cells were stained with APC-CD138 Ab and PE-B220 Ab (1:100 dilution) or isotype IgG for 30 min at 4 °C. Cells were washed with PBS 3 times and analyzed with a flow cytometer (FACSCanto II, BD Bioscience). The data were analyzed with Flowjo (Tree Star Inc., Ashland, OR) with the data of isotype IgG staining as a gating reference.

**Detection of apoptotic specific plasma cells**

B cells were cultured in the presence of CoV (10 µg/ml) for 24 h. One million cells per sample were harvested, washed and labelled with AF647-B220 Ab, AF700-CD138 Ab, pacific blue-GL7 Ab, propidium iodide (PI) and the AF488-Annexin V reagent kit following the manufacturer’s instruction. Cells were analyzed with a flow cytometer (BD FACSCanto II). CD138^+^ B220^+^ PCs were gated first, from which GL7^+^ PCs (GL7 is an activation marker of B cells; the GL7^+^ PCs are regarded as activated sPCs) were gated; then, The Annexin V^+^ or PI^+^ Annexin V^+^ cells were regarded as apoptotic cells.

**Isolation plasma cells from the spleen**

Spleen single cells were prepared as described above or collected from relevant experiments, and labeled with antibodies of AF647-B220 and AF700-CD138. The CD138^+^ B220^+^ plasma cells were sorted out by FACS. To isolated antigen-specific PCs (sPC), spleen cells were prepared from CoV-immunized mice, and incubated in the presence of CoV (1 µg/ml) overnight. Pacific blue-GL7^+^ (activation indicator of B cells) B220^+^ CD138^+^ cells were sorted by FACS and used as sPCs. Cell purity was greater than 97% as re-analyzed by FACS with the post-sort cells. Post-sort plasma cells were cultured immediately for further experiments.

**Cell culture**

Following established procedures {Nguyen, 2018 #30}, plasma cells were cultured in 96-well flat-bottom cell culture plates at 5 × 10^5^ cells/well, 200 μl/well, in RPMI 1640 medium supplemented with 10% fetal calf serum, streptomycin 0.1 mg/ml, penicillin 100 U/ml, 5 × 10^−5^ M 2-β-mercaptoethanol, CD40L (20 ng/ml) and 2 mM glutamine in humidified atmosphere of 5% CO_2_ at 37°C.

**RNA sequencing (RNAseq)**

Plasma cells were cultured in the presence of OVA (10 µg/ml) for 48 h. The cells were harvested at the end of culture. Total RNAs were extracted from CoV-activated sPCs with the TRIzol reagents following the manufacturer’s instruction. RNAs were sent to a professional company (YiGene Biotech, Shenzhen, China). The RNAseq and data analysis were carried out by professional staff in the company. Briefly, quality was verified using an RNA integrity number cut-off of 8. Poly-A enriched sequences were reversely transcribed, fragmented, and amplified using the SMARTer Universal Low RNA Kit (Clontech, Mountain View, CA). Sequencing was

performed using Hiseq 4000 (Illumina, San Diego, CA), 100 base-pair reads (paired-end).

**Plasma cell protein extraction**

Plasma cells were collected from relevant experiments and lysed with a lysis buffer (1.5 mM MgCl_2_; 10 mM HEPES; 0.5 mM DTT; 10 mM KCl; 0.05% NP40; 1 mM EDTA and protease inhibitor cocktail). Lysates were centrifuged at 13,000 *g* for 10 min. Supernatant was used as the cytosolic proteins. Pellets were resuspended in a nuclear lysis buffer (0.2 mM EDTA; 1.5 mM MgCl_2_SO_4_; 4.6 M NaCl; 5 mM HEPES; 0.5 mM DTT; 26% glycerol) and incubated for 30 min. Lysates were centrifuged at 13,000 *g* for 10 min. Supernatant was used as the nuclear proteins. All the procedures were performed at 4 °C.

**Western blotting**

Proteins (50 µg/well) were fractioned by SDS-PAGE (sodium dodecyl sulphate-polyacrylamide gel electrophoresis) and transferred onto a PVDF (polyvinylidene fluoride) membrane. The membrane was incubated with 5% skim milk in PBS for 30 min to block non-specific binding, incubated with primary Abs (see figures for Ab types; diluted to 1:500), washed with TBST (Tris-buffered saline containing 0.05% Tween 20) 3 times, incubated with HRP-labeled secondary Abs (diluted to 1:5,000) for 2 h at ambient temperature, washed with TBST 3 times. Immunoblots on the membrane were developed using the enhanced chemiluminescence and photographed in an image station (GelDoc Go image station; Bio-Rad).

**ROS detection by flow cytometry in plasma cells**

We used CellROX Deep Red Reagent to detect intracellular ROS in plasma cells, which freely diffuses into the cells to be oxidized by ROS and becomes highly fluorescent {Grinberg, 2012 #2}. Plasma cells were incubated with 1 μM CellROX Deep Red Reagent at 37 °C for 30 min, and the cells were analyzed by FACS.

**Co-immunoprecipitation (co-IP)**

Proteins were extracted from plasma cells as described above, incubated with protein G agarose beads for 2 h to adsorb pre-existing immune complexes. The beads were removed by centrifugation at 5,000 *g* for 5 min. Supernatant was incubated with Abs (1 µg/ml) of anti-pSTAT1 or anti-SOD or isotype IgG overnight; the samples were incubated with protein G agarose beads for 2 h to precipitate the immune complexes; the beads were collected by centrifugation at 5,000 *g* for 5 min. Proteins on beads were eluted with an eluting buffer (pH 8.5, 10 mM Tris-Cl) and analyzed by Western blotting to determine the protein levels of pSTAT1 and SOD.

**Detection of SOD ubiquitination**

The staining of complex of pSTAT1/SOD in the membrane was stripped off with a stripping solution (25 mM glycine-HCl, pH 2, 1% (w/v) SDS) and re-blotted using anti-ubiquitin Ab.

**Mass spectrometry analysis**

Protein samples were prepared with sPCs and re-suspended with Nano-RPLC buffer A. The samples were loaded on C18 nanoLC trap column (100 µm×3 cm, C18, 3 µm, 150 Å) and washed by Nano-RPLC Buffer A (0.1% FA, 2% ACN) at 2μl/min for 10 mins. An elution gradient of 5-35% acetonitrile (0.1% formic acid) in 90 mins gradient was used on an analytical ChromXP C18 column (75 μm x 15 cm, C18, 3 μm 120 Å) with spray tip. Data acquisition was performed with a Triple TOF 5600 System (AB SCIEX, USA) fitted with a Nanospray III source (AB SCIEX, USA) and a pulled quartz tip as the emitter (New Objectives, USA). Data were acquired using an ion spray voltage of 2.5 kV, curtain gas of 30 PSI, nebulizer gas of 5 PSI, and an interface heater temperature of 150 °C. For information dependent acquisition (IDA), survey scans were acquired in 250 ms and as many as 35 product ion scans were collected if they exceeded a threshold of 150 counts per second (counts/s) with a 2+ to 5+ charge-state. The total cycle time was fixed to 2.5 s. A rolling collision energy setting was applied to all precursor ions for collision-induced dissociation (CID). Dynamic exclusion was set for ½ of peak width (18 s). And the precursor was then refreshed off the exclusion list. Based on combined MS and MS/MS spectra, proteins were identified based on 95% or higher confidence interval of their scores in the MASCOT V2.3 search engine (Matrix Science Ltd., London, U.K.), using the following search parameters: cow- lacbobacillus casel mix database. trypsin as the digestion enzyme.

**Statistics**

The data are presented as mean ± SEM. Each sample was tested in triplicate (with the average of 3 readouts to be one datum). Each experiment was repeated 6 times. In animal experiments, each group consists of 6 mice. The difference between two groups was determined by the Student *t*-test. ANOVA followed by the Dunnett’s test or Bonferroni test was performed for multiple comparisons. The correlation between two groups was determined by the Pearson correlation coefficient test. P<0.05 was set as a significant criterion.


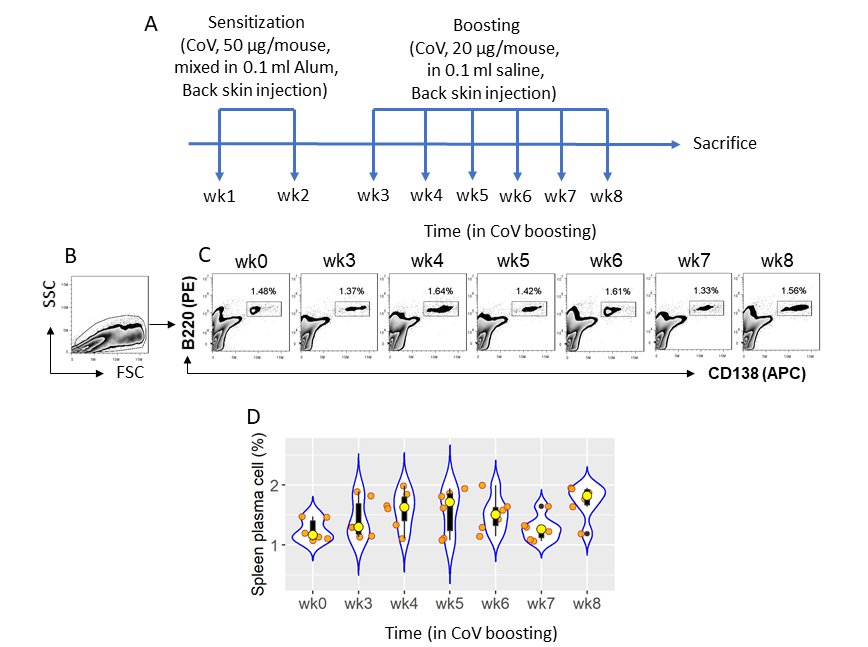


**Figure S1. Assessment of plasma cells in mouse spleen during immune boosting with CoV**. A, C57BL/6 mice were immunized with CoV/Alum on week (wk)1 and wk2; followed by Boosting with CoV once a week for 6 consecutive weeks. Spleen cells were prepared from mice at the timepoints denoted in figure and analyzed by FACS. B, the FSC/SSC plots were gated first. C, gated FACS plots show plasma cell frequency. D, violin plots show summarized plasma cell counts. Each group consists of 6 mice. The data of violin plots are presented as median (IQR). Each dot in violin plots presents data obtained from one mouse.


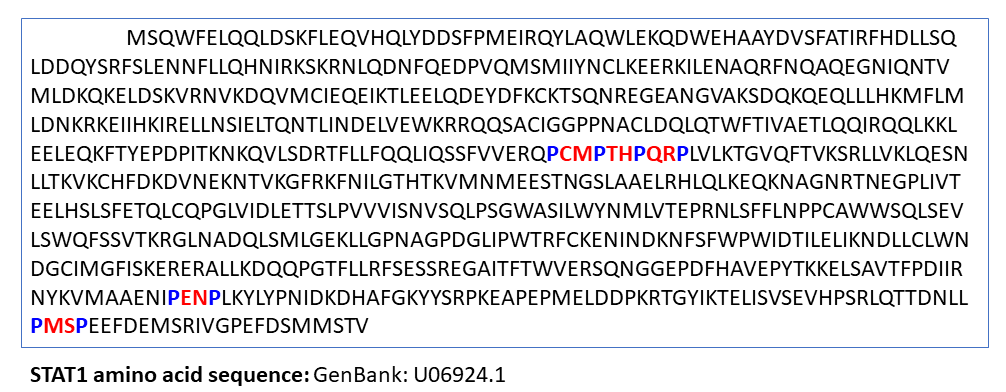


**Figure S2. “PxxP” structures in STAT1 amino acid sequence**. Five PxxP structures (labeled as blue and red color) in the amino acid sequence of STAT1 molecule.


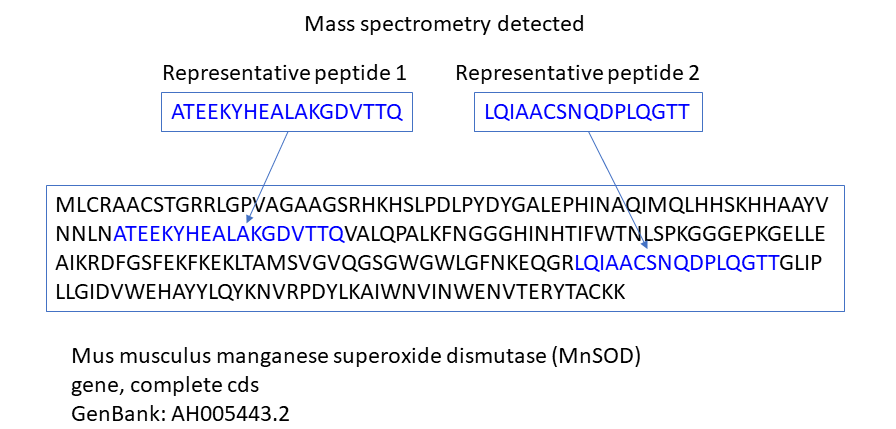


**Figure S3. Identification of SOD in a protein complex precipitated with anti-STAT1 Ab**. B cells were isolated from the naive mouse spleen and exposed to OVA (10 µg/ml) in the culture for 48 h. The protein extracts of B cells were precipitated with anti-pSTAT1 antibodies. The precipitated protein complexes were analyzed by mass spectrometry (MS). The MS results showed that, besides finding pSTAT1 protein in the complexes, the SOD protein was also found. Two representative peptides (in blue color) are presented that are pointed at the identical peptides in the SOD molecular amino acid sequence.


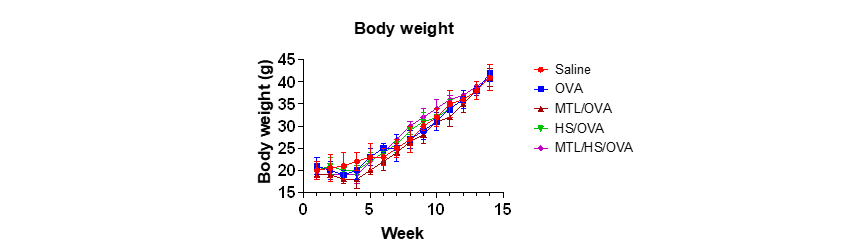


**Figure S4. Mouse body weight records**. Mouse body weight was recorded weekly throughout the entire experimental period (14 weeks). The curves show mouse body weight records in indicated groups. Each group consists of 6 mice. No statistical difference between groups.


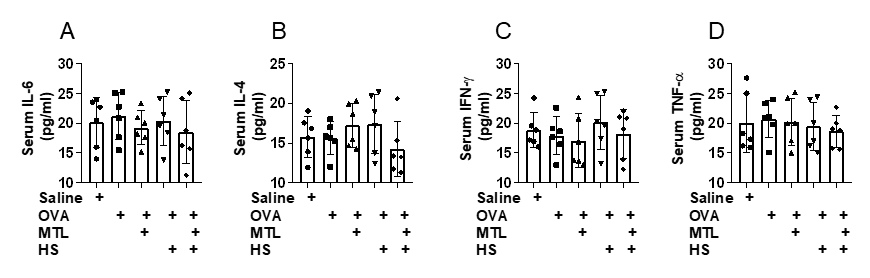


**Figure S5. Serum levels of representative inflammatory cytokines in mice**. C57BL/6 mice were immunized with OVA plus MTL or/and HS. Control mice were treated with saline. Mice were sacrificed at the end of experiments (14 weeks). Blood samples were collected from each mouse. The serum was isolated from the samples and analyzed by ELISA. The bars show the serum levels of IL-6 (A), IL-4 (B), IFN-γ (C) and TNF-α (D), respectively. The data were analyzed by ANOVA; no statistical difference was found between groups. Each group consists of 6 mice.


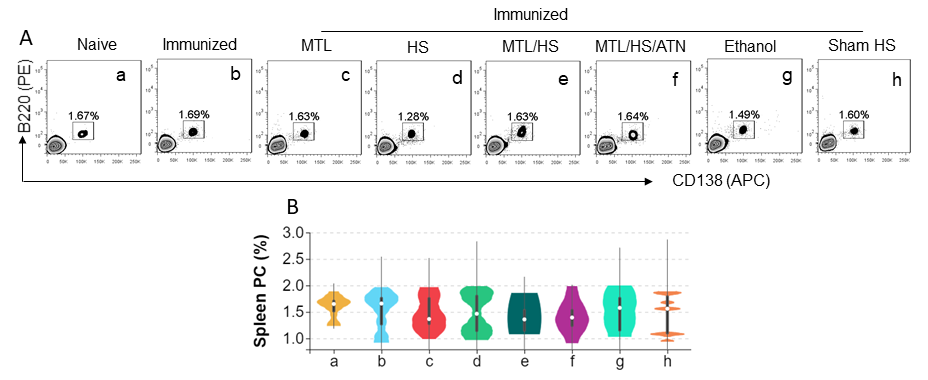


**Figure S6. Assessment of PC in the mouse spleen cells**. PCs were isolated from the spleen of immunized mice and treated with conditions in the culture as denoted in the figures. CoV: 10 µg/ml. MTL: 1% Maotai liquor (contains 1% alcohol) in culture medium. HS: Heat stress by placing cell culture vials in a water bath with indicated temperature for 1 h. Ethanol: 1%. ATN: ATN224 (a SOD1 inhibitor; 3.5 µM). A, gated FACS plots show PC frequency in spleen cells. B, violin plots show summarized PC counts in spleen cells. The data represent 6 independent experiments. No statistical difference was detected between groups (ANOVA followed by Dunnett’s test).


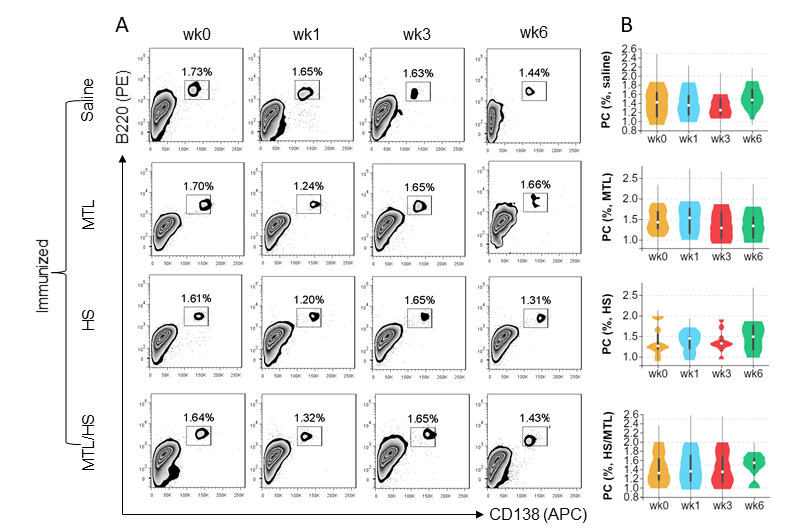


**Figure S7. Assessment of spleen PCs in mice after CoV-immunization**. Mice were immunized with CoV followed by CoV boosting with or without the treatment of MTL or/and HS as described in the text. Mice were sacrificed in wk0, or wk1, or wk3, or wk6, after the last CoV boosting. Spleen cells were prepared from each mouse and analyzed by FACS. A, gated FACS plots show PC frequency in spleen cells. B, violin plots show summarized PC counts. No statistical difference was detected between groups (ANOVA followed by Dunnett’s test).

Data of RNAseq

Gene id MeanTPM (B1) MeanTPM (A1) log2FoldChange GeneName

ENSG00000099194 57.428291 462.904358 2.0988532 TNFRSF1A

ENSG00000198804 13414.90332 19231.93945 3.7180993 TRADD

ENSG00000169710 57.824951 266.338776 0.0705675 TADD

ENSG00000166710 426.819214 2186.136963 1.9482458 DASP8

ENSG00000198712 20704.09961 27329.67383 3.5651429 FASLG

ENSG00000198938 10844.35742 15390.25098 0.8081824 FASLG

ENSG00000106366 933.928223 401.571472 2.7986534 P53

ENSG00000075624 8167.384277 6160.925781 2.042282 CASP3

ENSG00000080824 1490.74707 833.296082 0.5242199 CASP7

ENSG00000035862 376.067505 109.827377 1.9471594 BAX

ENSG00000241978 186.046173 64.20443 3.9628509 BAK1

ENSG00000108518 2504.897461 1388.552734 3.2418049 CYCS

ENSG00000134824 85.891846 364.775909 0.6001797 APAF1

ENSG00000146674 484.604004 148.506348 0.4711396 CASP9

ENSG00000117525 576.92981 211.557404 -3.45127 TWIST1

ENSG00000100316 1326.157837 2252.099609 0.9138565 BCL2

ENSG00000116133 137.232361 328.76181 -1.1114693 LIVIN

ENSG00000152291 14.166297 84.070442 -1.0930929 SURVIVIN

ENSG00000233276 191.74382 864.473572 -1.3420745 MCL1

ENSG00000234745 2010.829956 803.159485 3.6001985 TNFRSF1A

ENSG00000111640 6207.870605 7678.270996 0.3290247 TRADD

ENSG00000005884 687.808899 399.477753 4.3518057 TADD

ENSG00000171867 335.406189 114.976173 2.5321945 DASP8

ENSG00000179010 45.739002 245.846619 4.8670239 FASLG

ENSG00000115414 1139.515991 850.864929 -0.3136067 FASLG

ENSG00000169429 168.593857 505.684753 0.2521826 P53

ENSG00000148773 25.299454 65.062614 1.2519031 CASP3

ENSG00000127022 439.93985 397.052734 2.4868883 CASP7

ENSG00000149591 1005.758972 510.110168 2.494611 BAX

ENSG00000109971 3483.251221 2462.868652 1.8022411 BAK1

ENSG00000125148 3060.27124 1760.902222 3.3483433 CYCS

ENSG00000169174 15.915906 97.372498 2.9279415 APAF1

ENSG00000124208 47.931549 182.16655 2.0749314 CASP9

ENSG00000130164 27.601425 104.257599 -2.33562 TWIST1

ENSG00000163430 499.168915 590.036743 -1.2496951 BCL2

ENSG00000174136 426.541412 239.611023 -2.1886331 LIVIN

ENSG00000062485 173.541168 59.844494 -1.6420527 SURVIVIN

ENSG00000108468 83.617363 252.514816 -1.7207184 MCL1

ENSG00000147872 301.443817 91.013885 3.3500066 TNFRSF1A

ENSG00000160014 220.01239 438.331299 0.4650533 TRADD

ENSG00000124201 76.054817 24.048372 4.6857083 TADD

ENSG00000186480 31.113529 154.090271 -0.332066 DASP8

ENSG00000165732 140.083527 49.980057 4.8514418 FASLG

ENSG00000114850 556.865967 313.815369 1.8709175 FASLG

ENSG00000072310 20.472984 117.774475 3.1726843 P53

ENSG00000115762 145.838211 59.105385 3.1270832 CASP3

ENSG00000183291 298.427032 41.779907 0.3235821 CASP7

ENSG00000063978 76.533035 23.843962 4.9150377 BAX

ENSG00000067225 2234.289062 2817.328613 -0.8018675 BAK1

ENSG00000167552 331.929474 660.571655 1.9049734 CYCS

ENSG00000198727 6838.503418 8138.277344 -0.6728272 APAF1

ENSG00000196611 208.450546 58.315407 2.2638553 CASP9

ENSG00000057019 459.119507 284.166412 -1.8932398 TWIST1

ENSG00000184640 131.729019 233.735352 -0.8937091 BCL2

ENSG00000113739 276.541962 154.894928 -1.9753307 LIVIN

ENSG00000128245 125.514534 311.392273 -2.6319054 SURVIVIN

ENSG00000162430 40.656658 110.862244 -2.9649233 MCL1

ENSG00000173166 30.660248 10.488063 2.947855 TNFRSF1A

ENSG00000168461 68.51107 15.953934 -0.2367868 TRADD

ENSG00000138071 313.668976 118.975624 2.665706 TADD

ENSG00000244687 210.523682 71.536255 1.9863771 DASP8

ENSG00000111206 171.885925 75.570686 3.2418941 FASLG

ENSG00000128422 354.803558 168.934036 2.055421 FASLG

ENSG00000123416 2963.419434 3681.729736 4.0774265 P53

ENSG00000206053 74.901024 147.680496 2.0085042 CASP3

ENSG00000164111 1054.844116 582.17627 0.8388801 CASP7

ENSG00000167658 2033.433838 2383.145508 1.598805 BAX

ENSG00000197045 61.417885 15.594294 4.2977578 BAK1

ENSG00000108821 68.407082 137.349197 -0.8000285 CYCS

ENSG00000143418 337.553131 180.927017 0.7099079 APAF1

ENSG00000108854 204.875275 66.673019 4.9269365 CASP9

ENSG00000085063 357.798096 162.543335 -2.867116 TWIST1

ENSG00000013588 78.078339 139.163757 -2.7800383 BCL2

ENSG00000196230 2572.255859 3031.231445 1.4080747 LIVIN

ENSG00000163520 67.165764 146.440796 -0.4874476 SURVIVIN

ENSG00000163814 109.197655 48.174587 -2.5456155 MCL1

ENSG00000134107 17.279608 73.258049 1.5272383 TNFRSF1A

ENSG00000284461 0.245543 29.269135 4.5464229 TRADD

ENSG00000198431 562.273499 339.558624 -0.0092587 TADD

ENSG00000237973 1103.246216 1532.128418 4.386503 DASP8

ENSG00000143742 120.516418 300.422913 4.7168488 FASLG

ENSG00000154710 65.069984 17.145008 3.2545138 FASLG

ENSG00000188643 621.964539 273.915619 -0.7144072 P53

ENSG00000211459 2700.133545 1886.154663 2.6562947 CASP3

ENSG00000152104 6.727319 20.803608 -0.4889721 CASP7

ENSG00000131389 19.476433 44.331619 1.9911527 BAX

ENSG00000151835 3.730408 13.122587 2.0713117 BAK1

ENSG00000166923 47.713112 17.036995 1.6800889 CYCS

ENSG00000198561 26.584162 63.609219 2.362182 APAF1

ENSG00000013297 165.484436 54.346077 0.1878374 CASP9

ENSG00000184292 60.305088 155.491806 -2.6659381 TWIST1

ENSG00000072110 720.824341 420.864441 0.7883272 BCL2

ENSG00000167460 862.291565 657.076416 -0.4550455 LIVIN

ENSG00000198911 66.491074 128.284378 1.0875954 SURVIVIN

ENSG00000108691 91.211845 320.283264 -1.7853803 MCL1

ENSG00000115758 361.265717 185.185074 4.0615013 TNFRSF1A

ENSG00000198886 9112.984375 10065.78125 2.6607219 TRADD

ENSG00000162909 495.000793 330.430634 -0.4700703 TADD

ENSG00000104368 197.184525 78.513184 1.0332455 DASP8

ENSG00000065802 33.900272 23.79048 0.7530914 FASLG

ENSG00000157227 208.848816 113.786331 -0.9379945 FASLG

ENSG00000160285 48.257607 109.089455 1.5560749 P53

ENSG00000110092 119.229439 56.213215 0.7721218 CASP3

ENSG00000091527 142.598267 66.753868 4.2858053 CASP7

ENSG00000162458 78.223045 28.361914 2.8911115 BAX

ENSG00000071127 473.204987 314.482849 1.6219324 BAK1

ENSG00000198786 2004.780029 2425.024658 1.8935686 CYCS

ENSG00000106105 391.816589 199.153091 4.6357503 APAF1

ENSG00000182718 5285.227539 5022.780273 -0.5789427 CASP9

ENSG00000044574 445.96283 304.15683 -0.8551189 TWIST1

ENSG00000119729 60.894535 18.838827 -2.3601517 BCL2

ENSG00000167601 730.537659 566.519775 -1.4224095 LIVIN

ENSG00000230551 2.963637 16.2346 -2.066057 SURVIVIN

ENSG00000167772 54.224869 1.724662 -0.4665959 MCL1

ENSG00000103353 15.981384 41.621258 0.7257034 TNFRSF1A

ENSG00000166801 17.022697 60.603775 0.2056063 TRADD

ENSG00000101255 196.905106 93.632469 -2.6096552 TADD

ENSG00000272398 182.69989 351.717041 -2.4000185 DASP8

ENSG00000090857 15.236511 43.31007 -1.6467469 FASLG

ENSG00000142798 35.097157 58.789295 1.3982599 FASLG

ENSG00000150753 508.38147 274.156036 1.0203972 P53

ENSG00000113712 103.865417 77.01915 -0.2073547 CASP3

ENSG00000206527 15.592864 43.667736 -0.858943 CASP7

ENSG00000110321 522.024902 366.732758 -0.4153824 BAX

ENSG00000166128 36.269829 27.832153 0.4246369 BAK1

ENSG00000135069 228.217514 76.451561 0.4438173 CYCS

ENSG00000112078 16.569696 41.650196 -2.9934474 APAF1

ENSG00000141510 55.973255 136.661423 -1.0316165 CASP9

ENSG00000132912 32.806389 73.772011 0.6333858 TWIST1

ENSG00000250021 1.289031 31.763424 4.6504054 BCL2

ENSG00000128510 126.164566 230.025284 2.1038273 LIVIN

ENSG00000253767 0.377166 15.744785 4.5193076 SURVIVIN

ENSG00000128050 103.534523 118.354233 3.2592214 MCL1

ENSG00000135404 1396.890015 866.762695 -1.4624671 TNFRSF1A

ENSG00000122861 208.816284 445.553711 0.9662904 TRADD

ENSG00000079459 152.828873 293.351532 -2.7475633 TADD

ENSG00000111669 1397.267212 1810.641235 1.9729538 DASP8

ENSG00000135074 33.333294 18.677935 0.4775509 FASLG

ENSG00000140416 626.18396 440.200226 -0.6662082 FASLG

ENSG00000070669 207.470047 89.307625 0.87053 P53

ENSG00000112414 70.655373 35.893436 1.416186 CASP3

ENSG00000278540 16.156683 47.614368 1.7250466 CASP7

ENSG00000210082 6367.265625 5462.280273 -1.5581888 BAX

ENSG00000238227 10.958774 44.520752 0.7761966 BAK1

ENSG00000101224 156.355209 235.133942 -1.2820471 CYCS

ENSG00000149485 68.528717 142.344269 -1.1538938 APAF1

ENSG00000184575 112.407555 65.548103 -0.3740377 CASP9

ENSG00000144381 410.564117 245.826233 4.9417141 TWIST1

ENSG00000146112 26.585464 104.264984 2.246135 BCL2

ENSG00000198959 41.401024 13.594575 4.6013142 LIVIN

ENSG00000097033 42.832722 21.79357 -0.2649624 SURVIVIN

ENSG00000103064 39.300743 15.282007 2.7915708 MCL1

ENSG00000204673 29.385996 85.897789 1.8583467 TNFRSF1A

ENSG00000137094 6.50739 24.296333 -2.933825 TRADD

ENSG00000184009 3137.14502 2593.871094 -2.6671481 TADD

ENSG00000164163 119.572556 60.216068 0.2533274 DASP8

ENSG00000149600 113.951248 41.254826 -2.6066509 FASLG

ENSG00000165029 0.363223 7.202948 0.7776663 FASLG

ENSG00000117318 173.660278 336.820923 -1.064566 P53

ENSG00000147065 531.770081 394.142334 1.1018733 CASP3

ENSG00000137310 117.108086 53.39497 -1.3022785 CASP7

ENSG00000133657 108.421608 56.223145 -2.1120983 BAX

ENSG00000083845 811.284546 1180.824951 0.9056347 BAK1

ENSG00000160216 38.994259 81.638977 1.411026 CYCS

ENSG00000204160 43.321148 16.129524 -1.9910979 APAF1

ENSG00000179218 1123.933716 812.173828 -1.9304616 CASP9

ENSG00000108175 30.183123 55.922314 2.1035068 TWIST1

ENSG00000239672 661.133484 350.514648 1.0218461 BCL2

ENSG00000096384 1110.862305 862.426514 2.6908119 LIVIN

ENSG00000113161 33.591911 76.117432 4.8559615 SURVIVIN

ENSG00000196305 190.895706 116.258614 0.8276405 MCL1

ENSG00000166508 166.264297 259.326874 1.5256221 TNFRSF1A

ENSG00000100097 4746.880859 5693.055664 0.8432851 TRADD

ENSG00000049759 180.610229 81.469826 -0.7106392 TADD

ENSG00000076067 23.116577 20.697454 -1.1825817 DASP8

ENSG00000185414 60.734764 19.284311 0.181214 FASLG

ENSG00000142541 1840.887939 2299.438721 -2.0849293 FASLG

ENSG00000168874 23.164 70.635956 -1.8734613 P53

ENSG00000110090 56.062012 24.176941 -0.4418751 CASP3

ENSG00000135631 30.295355 55.490868 0.7047017 CASP7

ENSG00000172780 3.346725 18.146599 -0.4569744 BAX

ENSG00000164054 339.136078 189.584702 -2.6789969 BAK1

ENSG00000101363 175.453918 56.517834 -1.2404758 CYCS

ENSG00000163739 64.196121 177.192673 1.0110949 APAF1

ENSG00000242028 19.27232 5.475611 -0.3153239 CASP9

ENSG00000143878 278.581635 166.885056 3.9041904 TWIST1

ENSG00000196497 50.67844 18.459557 -0.9988166 BCL2

ENSG00000117632 913.984314 1110.119995 2.6674621 LIVIN

ENSG00000166147 16.758408 33.957428 2.9009334 SURVIVIN

ENSG00000108829 528.843567 370.405823 2.2713391 MCL1

ENSG00000145860 26.696716 73.164253 1.8384739 TNFRSF1A

ENSG00000119242 22.583658 74.366371 -0.7332538 TRADD

ENSG00000117394 83.681679 142.050705 0.6187576 TADD

ENSG00000006327 829.551025 522.997498 0.1012143 DASP8

ENSG00000171314 644.063965 842.790466 0.5556089 FASLG

ENSG00000204262 32.564125 61.038567 1.1885693 FASLG

ENSG00000135318 227.962387 136.887161 1.9027566 P53

ENSG00000181061 186.408478 81.598427 0.3564903 CASP3

ENSG00000127528 180.816437 90.222473 -1.9362852 CASP7

ENSG00000076248 18.9128 62.840721 -2.1092378 BAX

ENSG00000156508 6420.247559 6852.34375 -1.2355054 BAK1

ENSG00000116649 333.229706 167.725128 0.418708 CYCS

ENSG00000113013 520.041382 371.993652 1.7482782 APAF1

ENSG00000074800 3140.578369 3457.303711 -2.3790403 CASP9

ENSG00000123485 62.32906 24.07527 2.9136816 TWIST1

ENSG00000113721 4.855587 22.640089 1.3095987 BCL2

ENSG00000171155 38.751724 3.226553 0.9111144 LIVIN

ENSG00000130402 830.238953 462.646637 -0.1575564 SURVIVIN

ENSG00000081923 123.860367 73.053429 -0.8533564 MCL1

ENSG00000139278 64.00956 34.681751 -0.1488115 TNFRSF1A

ENSG00000178974 24.794485 5.079003 -0.689058 TRADD

ENSG00000272325 9.600573 1.910498 -1.1411893 TADD

ENSG00000228253 5237.90332 3617.833984 0.3757526 DASP8

ENSG00000162139 11.08221 3.773757 -0.6269247 FASLG

ENSG00000024422 108.345451 58.179508 1.5588623 FASLG

ENSG00000130479 38.776272 20.784891 0.5574399 P53

ENSG00000178209 158.954391 123.67643 -2.684421 CASP3

ENSG00000283239 0.064763 8.947206 -1.7072566 CASP7

ENSG00000138443 52.511433 48.038109 -2.43827 BAX

ENSG00000134590 70.24897 171.263077 -2.929075 BAK1

ENSG00000270181 0.021933 13.655273 -0.5041362 CYCS

ENSG00000132561 38.527443 80.568764 1.6209013 APAF1

ENSG00000074416 100.329239 61.464348 -0.0043544 CASP9

ENSG00000139112 8.193023 36.72686 4.8246563 TWIST1

ENSG00000205542 4745.946289 3916.798096 4.7168049 BCL2

ENSG00000129473 23.866175 9.085585 4.0509456 LIVIN

ENSG00000075426 58.288162 90.097679 2.0298675 SURVIVIN

ENSG00000128272 711.864685 528.887573 0.0486155 MCL1
